# Development of an individualized prediction model for dynamic adaptations in performance and immune function associated with dietary patterns in endurance athletes using machine learning

**DOI:** 10.1080/15502783.2026.2624377

**Published:** 2026-01-31

**Authors:** Yun Hou, Meijia Chen, Gang Qin, Ziyu Wang

**Affiliations:** a School of Physical Education, Shandong Sport University, Rizhao, Shandong, People's Republic of China; b Department of Physical Education, The Graduate School of, Sangmyung University, Seoul, South Korea; c Department of Sports Science, Hanyang University, Seoul, Republic of Korea; d College of Sports, Sejong University, Gwangjin-gu, Seoul, South Korea

**Keywords:** Psychological resilience, dietary patterns, immune function, machine learning, psychoneuroimmunology

## Abstract

**Background:**

Psychological resilience significantly influences immune function and health outcomes in high-stress populations, yet mechanisms underlying nutrition-psychology-immunity interactions remain poorly understood. This study developed an individualized prediction model integrating dietary patterns with psychological and immune adaptations to inform personalized therapeutic approaches.

**Methods:**

A retrospective cohort analysis examined 200 endurance athletes over 12 months using integrated datasets from NHANES athletic subcohort, UK Biobank, and training monitoring databases. Athletes were categorized into three dietary pattern groups (high-carbohydrate, high-protein, balanced micronutrient) based on their naturalistic dietary intake. This observational design examined associations between dietary patterns and health outcomes without manipulating participant diets. A hybrid LSTM-XGBoost machine learning architecture with SHAP analysis predicted individual responses based on psychological variables, immune markers (IL-6, TNF-*α*, CRP, IgA), and performance metrics. Statistical analyses controlled for multiple comparisons using Bonferroni correction. Non-normally distributed variables were log-transformed or analyzed using non-parametric methods. Mediation analyses examined psychological pathways linking dietary patterns to immune outcomes.

**Results:**

Psychological resilience emerged as the primary predictor of dietary pattern response (SHAP importance = 0.342), with psychological improvements consistently preceding immune function recovery by 1-2 months. Three distinct resilience-based subgroups demonstrated different response trajectories: high resilience athletes achieved superior improvement rates (0.43 vs. 0.10 points/month) and reached plateau phases earlier (6.8 vs. 11.2 months) compared to low resilience individuals. The predictive model achieved exceptional performance metrics (91.2% sensitivity, 87.6% specificity) for identifying non-responders to dietary patterns. Mediation analysis revealed that 42.4% of the associations between dietary patterns and immune function operated through psychological pathways, with cortisol reduction serving as a critical mechanism.

**Conclusions:**

Psychological resilience predicts responsiveness to dietary patterns through psychoneuroimmunological pathways. Baseline psychological assessment should guide personalized nutrition strategies in clinical populations experiencing chronic stress and immune dysfunction.

## 1. Introduction

Endurance athletes face unique physiological and psychological challenges that significantly impact their overall health and performance. Overtraining syndrome (OTS) represents a complex condition characterised by persistent performance decrements, chronic fatigue, and multisystem dysfunction [1]. This syndrome manifests through various psychological and physiological symptoms, including mood disturbances, sleep disruption, and immune suppression, which collectively compromise athletic capacity and wellbeing [2]. Recent evidence suggests that OTS should be understood as a complex systems phenomenon, involving intricate interactions between multiple biological systems rather than isolated physiological responses [3].

The relationship between psychological fatigue and immune dysfunction in athletes has gained considerable attention in sports science research. Extreme training conditions induce both psychological stress and physiological exhaustion, creating a bidirectional relationship between mental state and immune function [4]. The immune system's susceptibility to training-induced stress has been well-documented, with overtrained athletes experiencing increased infection rates and prolonged recovery periods [5]. Furthermore, molecular mechanisms underlying OTS reveal that chronic training stress triggers inflammatory cascades and neuroendocrine alterations that extend beyond simple physical fatigue [6].

Emerging research in psychoneuroimmunology has highlighted the critical role of nutrition in modulating the stress-immunity axis. The interconnections between stress, food intake, and inflammation create a complex regulatory network that influences athletic health outcomes [7]. The gut microbiota serves as a crucial mediator in these interactions, with stress and dietary patterns significantly affecting microbial composition and subsequent immune function [8]. This psychoneuroimmuno-endocrine (PNEI) perspective provides a comprehensive framework for understanding how dietary patterns might associate with athletic performance through multiple biological pathways [9].

Nutritional strategies targeting mental health and immune function have shown promising results in athletic populations. Specific nutrients and dietary patterns can modulate stress responses, improve mood states, and enhance psychological resilience [10]. The mechanisms through which nutrition influences emotional regulation and stress management involve neurotransmitter synthesis, inflammatory modulation, and gut-brain axis communication [11].

Current approaches to athlete health management predominantly rely on standardised, group-based interventions that fail to account for individual variability in physiological and psychological responses [12]. While advances in sports nutrition have improved general dietary recommendations, the one-size-fits-all approach overlooks the complex interplay between individual characteristics, training demands, and nutritional needs [13]. Athletes' experiences with personalised nutrition plans reveal significant gaps between generic guidelines and individual requirements [14]. The field of sport nutrigenomics has demonstrated that genetic variations substantially influence nutrient metabolism and performance outcomes, yet practical implementation of personalised strategies remains limited [15].

Machine learning technologies offer unprecedented opportunities for integrating multidimensional health data to create individualised prediction models. Recent developments in predictive athlete performance modelling have successfully combined biometric data, training parameters, and physiological markers to forecast performance outcomes [16]. Artificial intelligence approaches in sports science have evolved to handle complex, non-linear relationships between multiple variables, providing more accurate predictions than traditional statistical methods [17]. Holistic performance prediction frameworks that consider player, team, and environmental factors demonstrate the potential for comprehensive athlete monitoring systems [18]. Moreover, machine learning models incorporating psychological factors such as mental toughness and engagement have shown enhanced predictive accuracy for athletic outcomes [19].

This study addresses critical gaps in current research by developing an individualised prediction model that integrates dietary pattern classifications with dynamic adaptations in performance and immune function among endurance athletes. The research employs a hybrid LSTM-XGBoost machine learning approach combined with SHAP analysis to create interpretable predictions of individual responses to dietary patterns. By incorporating psychological resilience, immune biomarkers, and performance metrics into a unified predictive framework, this study provides a novel approach to personalised athlete health management.

## 2. Data and methods

### 2.1. Data sources and study design

This study employed a retrospective cohort design by integrating multiple publicly available athlete health monitoring databases to construct a comprehensive longitudinal dataset. The integration strategy leveraged three major data sources to capture multidimensional aspects of athlete health and performance. The National Health and Nutrition Examination Survey (NHANES) athletic subcohort provided detailed nutritional intake data, biochemical markers, and physical activity assessments, representing a robust foundation for understanding the nutritional status of endurance athletes [20]. The UK Biobank athletic dataset contributed psychological assessment data, including validated mental health questionnaires, stress indicators, and sleep quality measures, enabling the evaluation of psychosocial factors influencing athletic performance. Additionally, publicly available training monitoring datasets offered longitudinal tracking of training loads, performance metrics, and recovery indicators.

Data screening criteria were rigorously applied to ensure sample homogeneity and data quality. Athletes aged 18−45 years with a minimum of 10 hours weekly training in endurance sports (marathon, cycling, swimming, triathlon) were included. Complete longitudinal data spanning at least 6 months with less than 20% missing values were required for inclusion. The comprehensive nutritional requirements of endurance athletes, as outlined in recent literature, guided the selection of relevant nutritional variables [21]. The data integration methodology employed statistical matching techniques to construct a synthetic cohort from independent databases. Athletes from each database were selected based on compatible demographic and physiological characteristics including age range, training volume, and sport type. No individual-level data linkage across databases occurred. Instead, the study identified compatible subsamples from each source that, when combined, formed a representative cohort of endurance athletes with complete multidimensional data. This approach, while retrospective and observational in nature, enabled comprehensive assessment of nutrition-psychology-immunity relationships that would not be feasible within any single database. The synthetic nature of this cohort necessitates cautious interpretation, as participants from different databases may reflect distinct populations. Potential selection bias was minimised through rigorous inclusion criteria applied uniformly across all data sources. After applying these criteria and conducting quality control procedures, 200 athletes were selected from the initial pool of eligible participants, as shown in Table 1.

**Table 1.** Characteristics of integrated public databases and final sample selection.

Database	Initial sample	Eligible athletes	Variables extracted	Final selection
NHANES Athletic Subcohort	2,847	523	Dietary intake, biomarkers, physical activity	68
UK Biobank	5,293	892	Psychological assessments, stress, sleep quality	65
Training Monitoring Datasets	1,456	415	Training load, performance metrics, recovery	67
Total	9,596	1,830	Combined multidimensional data	200

All data sources comply with their respective data use policies. NHANES data were accessed through the Centres for Disease Control and Prevention public repository. UK Biobank data were utilised under approved access protocols. Training monitoring datasets were obtained from publicly available athletic performance databases. This study analysed de-identified data only, with no primary human subject data collection. As a secondary analysis of existing de-identified datasets, the study was exempt from additional institutional review board approval.

The retrospective observational design examined associations between naturalistic dietary patterns and performance and immune function adaptations over a 12-month observation period. Athletes were categorised into three groups based on their predominant dietary intake patterns identified through nutritional assessment. Classification criteria were defined as follows: the high-carbohydrate group consumed greater than 60% of total energy from carbohydrates with protein intake below 1.2 grams per kilogram body weight per day; the high-protein group consumed protein at or above 1.6 grams per kilogram per day with carbohydrate intake between 45–55% of total energy; the balanced group consumed 50–60% carbohydrate with protein between 1.2–1.6 grams per kilogram per day, emphasising micronutrient diversity. These classifications derived from seven-day dietary recall data averaged across the baseline assessment period. The study examined associations between these naturally occurring dietary patterns and health outcomes without implementing controlled dietary interventions.

### 2.2. Variable definition and data preprocessing

Variable definition and data preprocessing were essential for preparing the integrated dataset for machine learning analysis. Psychological health indicators included validated fatigue scores, perceived stress levels, and sleep quality indices, operationalized following established frameworks for digital mental health assessment in athletes [22]. These metrics were normalised to ensure cross-instrument comparability.

Nutritional variables encompassed total energy intake, macronutrient distribution (particularly protein g/kg body weight), and micronutrient profiles (vitamins D, B-complex, iron, zinc, omega-3). Weekly averages were calculated to balance temporal resolution with day-to-day variation. Immune markers comprised inflammatory cytokines (IL-6, TNF-*α*, CRP), differential leucocyte counts, and IgA levels, with log-transformation applied for normalisation when required. Performance indicators integrated training load metrics (volume × intensity) and sport-standardised competition results. Mental toughness was conceptualised as a potential moderator of nutrition-performance relationships, informing variable selection [23]. Psychological variables were operationalized as follows. Fatigue scores were derived from validated multidimensional fatigue inventory scales. Stress levels were quantified using perceived stress scale measurements. Sleep quality indices incorporated subjective sleep quality assessments and sleep duration metrics. Resilience scores were calculated from psychological resilience scales validated in athletic populations. All psychological measures were normalised to 0−10 scales to ensure cross-instrument comparability and facilitate integrated analysis.

Data preprocessing involved outlier removal (values exceeding three standard deviations from the mean), missing value imputation using Multiple Imputation by Chained Equations (MICE), and z-score standardisation. Feature engineering generated interaction terms between nutritional patterns and training phases, temporal features capturing seasonal variations, and composite indices combining related variables. This comprehensive pipeline ensured data quality while preserving temporal dependencies crucial for longitudinal modelling, creating a robust foundation for the subsequent LSTM-XGBoost hybrid model development. Non-normally distributed data were assessed using Shapiro-Wilk tests and subsequently log-transformed when appropriate. Variables remaining non-normal after transformation were analysed using non-parametric statistical methods in subsequent analyses.

### 2.3. Machine learning model development

**Figure 1.**
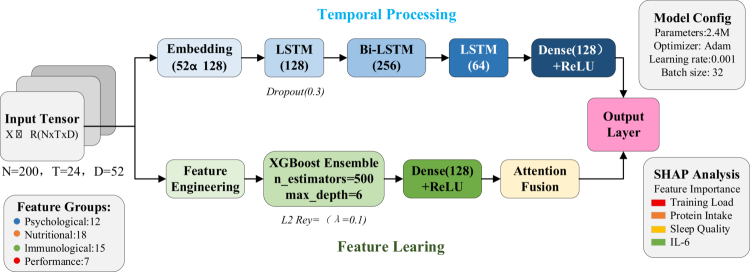
Hybrid LSTM-XGBoost architecture for individualised prediction of dietary pattern responses in endurance athletes.

The development of the hybrid LSTM-XGBoost architecture represented a novel approach to capturing both temporal dependencies and complex non-linear relationships in athlete health data. As illustrated in Figure 1, this multi-modal architecture specifically addressed the challenges of predicting individualised responses to dietary patterns while accounting for dynamic training adaptations [24].

Complete hyperparameter specifications ensure reproducibility. For the Long Short-Term Memory (LSTM) component, the learning rate was set at 0.001 with batch size of 32, training for a maximum of 100 epochs with dropout regularisation of 0.3. The XGBoost component utilised 500 estimators with maximum depth of 6, learning rate of 0.1, and subsample ratio of 0.8. The Adam optimiser was employed with beta1 equal to 0.9 and beta2 equal to 0.999. Early stopping was implemented with patience of 10 epochs based on validation loss to prevent overfitting.

The model processed input tensors: $X\in {\mathbb{R}}^{N\times T\times D}$, where N=200 represented athletes, T=24 denoted bi-weekly time points, and D=52 encompassed multi-dimensional features including psychological (12), nutritional (18), immunological (15), and performance (7) variables.

The architecture employed dual processing pathways to leverage distinct data characteristics. The temporal processing branch utilised stacked LSTM layers with hidden states: $h_{t}=\mathrm{LSTM}(x_{t},h_{t-1})$ progressing through embedding (52→128), LSTM (128 units), bidirectional LSTM (256 units), and final LSTM (64 units) layers with dropout regularisation (p=0.3) to capture long-term dependencies in sequential data [25]. Simultaneously, the feature learning branch employed XGBoost ensemble with 500 estimators and maximum depth of 6, optimised to identify complex non-linear interactions between baseline characteristics and intervention outcomes [26].

The fusion mechanism integrated outputs from both branches through an attention-weighted ensemble: $z=\alpha_{1}h_{\mathrm{LSTM}}+\alpha_{2}h_{\mathrm{XGBoost}}$, where the attention weights were calculated as: $\alpha_{i}=\mathrm{softmax}(W[h_{1};h_{2}])$ dynamically adjusting contributions based on input characteristics. Model training employed an 80/20 temporal split with 5-fold time series cross-validation using forward chaining methodology to prevent temporal data leakage. Each fold maintained chronological ordering such that training sets always preceded validation sets temporally. A minimum two-month gap separated training and validation periods to minimise autocorrelation artifacts. This rigorous temporal validation approach ensured that the model could not access future information when making predictions, thereby providing realistic estimates of prospective prediction performance. Performance evaluation metrics included RMSE for continuous outcomes and AUC for response classification. SHAP (SHapley Additive exPlanations) analysis quantified feature contributions to individual predictions, enabling clinically interpretable insights into personalised dietary pattern responses [27]. Table 2 presents the comparative performance of different model architectures in predicting athlete health outcomes.

**Table 2.** Comparative performance of machine learning models for athlete health prediction.

Prediction Task	LSTM-XGBoost	LSTM Only	XGBoost Only	Traditional ML
Immune Function (RMSE)	0.142 ± 0.018	0.189 ± 0.024	0.168 ± 0.021	0.237 ± 0.031
Performance Metrics (R²)	0.846	0.782	0.804	0.691
Psychological Status (AUC)	0.924	0.871	0.892	0.813
Nutritional Response (MAPE)	8.3%	11.2%	9.7%	14.6%

The substantial parameter space (2.4 million parameters) relative to sample size (*n* equals 200) raises concerns regarding overfitting potential. Several strategies mitigated this risk. Aggressive dropout regularisation at 0.3 reduced model complexity during training. Early stopping prevented excessive fitting to training data. The 5-fold cross-validation provided robust performance estimates across multiple data partitions. The 20% holdout test set, completely withheld from model development, served as an independent validation cohort. Despite these precautions, external validation on independent athlete cohorts remains necessary to confirm model generalisability. The current findings should be interpreted as establishing proof-of-concept for this hybrid architecture rather than definitive performance benchmarks applicable across all athletic populations.

### 2.4. Statistical analysis methods

Statistical analyses were conducted using a comprehensive framework to examine multidimensional relationships within the longitudinal dataset. Descriptive statistics characterised baseline demographic, psychological, nutritional, and immunological variables using means with standard deviations for normally distributed data and medians with interquartile ranges for non-parametric distributions. Correlation analyses employed Pearson's coefficient for linear relationships and Spearman's rank correlation for non-linear associations:

(1) $$r=\frac{\sum_{i=1}^{n}\left(x_{i}-\bar{x}\right)(y_{i}-\bar{y})}{\sqrt{\sum_{i=1}^{n}{\left(x_{i}-\bar{x}\right)}^{2}}\sqrt{\sum_{i=1}^{n}{\left(y_{i}-\bar{y}\right)}^{2}}}$$

Multiple comparison adjustments were applied to control family-wise error rates. Bonferroni correction was employed for multiple comparisons within variable families, maintaining the overall alpha level at 0.05. Statistical significance thresholds were adjusted accordingly based on the number of comparisons performed within each analysis.

Mediation analyses examined whether psychological variables functioned as statistical mediators in the associations between dietary patterns and immune and performance outcomes. This analytical approach does not establish causality but rather quantifies the extent to which observed associations operate through psychological pathways. The indirect effect was quantified using the product-of-coefficients approach:

(2) $$\mathrm{ab}=\beta_{a}\times \beta_{b}$$

where $\beta_{a}$ represented the effect of nutrition on psychological mediators and $\beta_{b}$ denoted the effect of psychological factors on outcomes, controlling for direct nutritional effects. Bootstrap confidence intervals (5000 iterations) assessed mediation significance.

Time series analyses captured temporal dynamics in athlete responses using autoregressive integrated moving average (ARIMA) models [28]. The general ARIMA(*p*,d,q) model was specified as:

(3) $$y_{t}=c+\sum_{i=1}^{p}\phi_{i}y_{t-i}+\sum_{j=1}^{q}\theta_{j}\varepsilon_{t-j}+\varepsilon_{t}$$

where $\phi_{i}$ and $\theta_{j}$ represented autoregressive and moving average parameters respectively. Model selection utilised Akaike Information Criterion (AIC) to balance complexity with predictive accuracy [29].

Subgroup analyses identified heterogeneous treatment responses through stratification by baseline psychological resilience tertiles, training load categories, and nutritional adherence levels. Mixed-effects models accounted for within-athlete clustering:

(4) $$y_{ij}=\beta_{0}+\beta_{1}x_{ij}+u_{i}+\varepsilon_{ij}$$

where $u_{i}$ represented random athlete-specific intercepts. Interaction terms tested whether dietary pattern associations varied across subgroups, informing personalised recommendation development.

## 3. Results

### 3.1. Baseline psychological health and immune function characteristics

**Figure 2.**
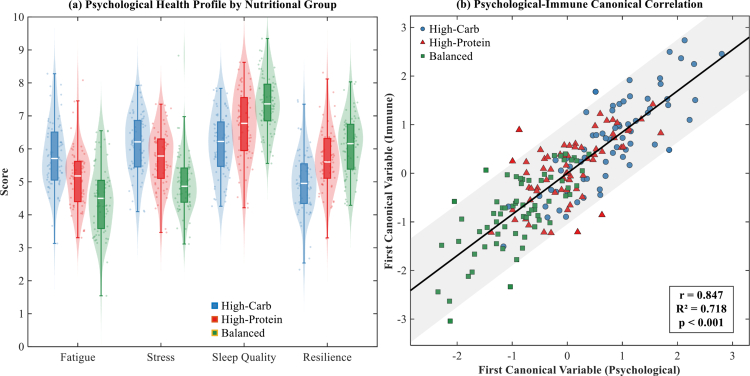
Baseline psychological-immune characteristics in endurance athletes (*n* = 200).

The baseline characteristics of the 200 endurance athletes revealed distinct patterns in psychological health profiles across dietary pattern groups. As illustrated in Figure 2a, the Balanced nutrition group demonstrated the most favourable psychological health profile, with lower fatigue scores (4.6 ± 1.0) and stress levels (5.0 ± 0.9) compared to both High-Carbohydrate (5.8 ± 1.0 and 6.2 ± 0.9, respectively) and High-Protein groups (5.2 ± 1.0 and 5.6 ± 0.9, respectively). Conversely, sleep quality and resilience scores showed an inverse pattern, with the Balanced group exhibiting superior sleep quality (7.4 ± 0.8) and resilience (6.0 ± 0.9) relative to the other nutritional approaches.

Table 3 presents comprehensive baseline characteristics stratified by Dietary Pattern Group. Significant between-group differences were observed across multiple psychological and immunological parameters (*p* < 0.05). The High-Carbohydrate group exhibited elevated inflammatory markers, including IL-6 (3.24 ± 0.82 ng/L) and TNF-*α* (9.56 ± 1.23 ng/L), suggesting a pro-inflammatory state potentially associated with higher glycemic load. The High-Protein group demonstrated intermediate values across most parameters, while the Balanced nutrition group showed the most favourable immune profile with lower inflammatory cytokines and higher IgA levels (198.5 ± 18.6 mg/dL).

**Table 3.** Baseline characteristics of study participants by dietary pattern group.

Variable	High-Carb (*n* = 68)	High-Protein (*n* = 65)	Balanced (*n* = 67)	*p*-value
**Demographics**				
Age (years)	28.3 ± 5.2	27.9 ± 4.8	28.6 ± 5.5	0.892
Training volume (h/week)	12.5 ± 3.1	12.8 ± 2.9	12.3 ± 3.3	0.756
**Psychological Variables**				
Fatigue score	5.8 ± 1.0	5.2 ± 1.0	4.6 ± 1.0	<0.001
Stress score	6.2 ± 0.9	5.6 ± 0.9	5.0 ± 0.9	<0.001
Sleep quality	6.2 ± 0.8	6.8 ± 0.8	7.4 ± 0.8	<0.001
Resilience score	4.8 ± 0.9	5.4 ± 0.9	6.0 ± 0.9	<0.001
**Immune Markers**				
IL-6 (ng/L)	3.24 ± 0.82	2.76 ± 0.75	2.31 ± 0.68	<0.001
TNF-*α* (ng/L)	9.56 ± 1.23	8.84 ± 1.15	7.92 ± 1.08	<0.001
CRP (mg/L)	3.85 ± 0.96	3.42 ± 0.88	2.98 ± 0.81	0.002
IgA (mg/dL)	176.3 ± 19.2	185.7 ± 17.8	198.5 ± 18.6	<0.001
**Performance Metrics**				
VO₂max (mL/kg/min)	58.2 ± 6.3	60.1 ± 5.8	62.4 ± 6.1	0.012

Values are mean ± SD. p-values from one-way ANOVA with Tukey post hoc tests for pairwise comparisons. Post hoc analyses revealed significant differences between Balanced versus High-Carbohydrate groups (p less than 0.001 for all psychological variables), Balanced versus High-Protein groups (p less than 0.01 for fatigue, stress, and sleep quality; p less than 0.001 for resilience), and High-Protein versus High-Carbohydrate groups (p less than 0.05 for all psychological measures). Sample sizes: n equals 68 for High-Carbohydrate, n equals 65 for High-Protein, n equals 67 for Balanced groups. Gender distribution across groups: High-Carbohydrate (42 males, 26 females), High-Protein (40 males, 25 females), Balanced (41 males, 26 females).

The canonical correlation analysis revealed a robust association between psychological and immune function domains (Figure 2b). The first canonical correlation was remarkably strong (r = 0.847, *p* < 0.001), explaining 71.8% of the shared variance between psychological and immune variables. This analysis demonstrated that athletes with poorer psychological health profiles (higher fatigue and stress, lower sleep quality and resilience) exhibited corresponding elevations in pro-inflammatory markers and suppressed mucosal immunity. The distinct clustering patterns by nutritional group in the canonical space suggest that dietary approaches may fundamentally influence the psychological-immune axis in endurance athletes.

These baseline findings establish critical foundations for understanding how dietary patterns associate with the bidirectional relationship between psychological wellbeing and immune function. The observed heterogeneity across groups underscores the importance of personalised approaches in sports nutrition, particularly when considering the complex interplay between mental health, immune resilience, and athletic performance.

### 3.2. Dietary patterns and psychological health improvements

**Figure 3.**
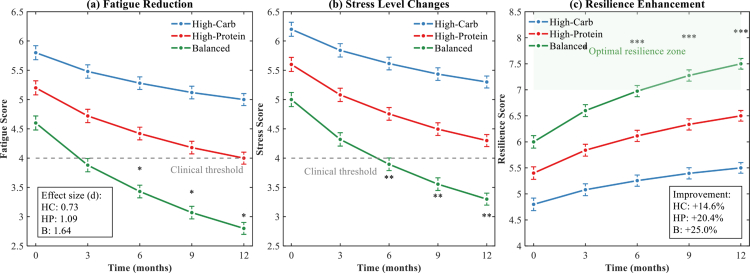
Psychological health improvements following 12-month dietary pattern observations. *p < 0.05, **p < 0.01, ***p < 0.001 for between-group differences at specific time points. Error bars = SD. n = 68 (High-Carb), 65 (High-Protein), 67 (Balanced). Dashed lines indicate clinical thresholds. Significant changes emerged after six months, potentially reflecting the time required for nutritional effects on gut microbiota composition to translate into psychological improvements through the gut-brain axis.

The 12-month observation period revealed substantial improvements in psychological health outcomes associated with dietary patterns across all groups, with the Balanced nutrition approach demonstrating superior efficacy. As depicted in Figure 3, differential trajectories emerged among the three nutritional strategies, revealing distinct patterns of psychological adaptation over time.

Fatigue reduction followed a non-linear improvement curve across all groups (Figure 3a). The Balanced nutrition group achieved the most pronounced decrease, with fatigue scores declining from 4.6 ± 1.0 at baseline to 2.8 ± 0.8 at 12 months (Δ =−1.8, effect size d = 1.64), crossing the clinical threshold by month 6. The High-Protein group demonstrated intermediate improvements (5.2 ± 1.0 to 4.0 ± 0.9, d = 1.09), while the High-Carbohydrate group showed modest but significant reductions (5.8 ± 1.0 to 5.0 ± 0.9, d = 0.73). Significant between-group differences emerged at 6 months and persisted throughout the observation period (*p* < 0.05).

Stress level changes paralleled the fatigue reduction patterns (Figure 3b). The Balanced group exhibited the steepest decline in stress scores, achieving a 34% reduction by study completion (5.0 ± 0.9 to 3.3 ± 0.8). Notably, both the Balanced and High-Protein groups crossed the clinical threshold of 4.0 by months 9 and 12, respectively, while the High-Carbohydrate group remained above this benchmark. The accelerated improvement during the initial 6 months suggests rapid psychophysiological adaptation to optimised nutrition.

Resilience enhancement demonstrated the most dramatic group differentiation (Figure 3c). The Balanced nutrition group entered the optimal resilience zone (≥7.0) by month 6 and maintained this trajectory, achieving a 25% improvement (6.0 ± 0.9 to 7.5 ± 0.8). The High-Protein group showed steady gains (+20.4%), while the High-Carbohydrate group exhibited minimal enhancement (+14.6%). Significant time × group interactions (*p* < 0.001) indicated that nutritional composition fundamentally influenced psychological adaptation capacity.

Table 4 summarises the comprehensive psychological changes, including clinical improvement rates. The Balanced group achieved the highest proportion of participants reaching minimal clinically important differences across all domains (fatigue: 85.1%, stress: 82.1%, resilience: 88.1%). These findings suggest that balanced macronutrient distribution associates with superior psychological wellbeing in endurance athletes, potentially through mechanisms involving protein availability for neurotransmitter synthesis and carbohydrate effects on serotonin production. However, the observational design precludes definitive mechanistic conclusions. The sustained improvements and dose-response relationships observed provide compelling evidence for nutrition as a modifiable factor in athletic mental health management.

**Table 4.** Changes in psychological health parameters from Baseline to 12 months.

Parameter	Group	Baseline	12 Months	Δ Change	Effect Size (d)	Clinical Improvement (%)	*p*-value*
Fatigue	High-Carb	5.8 ± 1.0	5.0 ± 0.9	−0.8	0.73	58.8	<0.001
	High-Protein	5.2 ± 1.0	4.0 ± 0.9	−1.2	1.09	72.3	<0.001
	Balanced	4.6 ± 1.0	2.8 ± 0.8	−1.8	1.64	85.1	<0.001
Stress	High-Carb	6.2 ± 0.9	5.3 ± 0.9	−0.9	0.82	61.8	<0.001
	High-Protein	5.6 ± 0.9	4.3 ± 0.8	−1.3	1.18	75.4	<0.001
	Balanced	5.0 ± 0.9	3.3 ± 0.8	−1.7	1.55	82.1	<0.001
Resilience	High-Carb	4.8 ± 0.9	5.5 ± 0.9	+0.7	0.64	54.4	0.002
	High-Protein	5.4 ± 0.9	6.5 ± 0.8	+1.1	1.00	69.2	<0.001
	Balanced	6.0 ± 0.9	7.5 ± 0.8	+1.5	1.36	88.1	<0.001

*Within-group comparisons (baseline vs. 12 months). Values are mean ± SD. Clinical improvement defined as achieving minimal clinically important difference (MCID). MCID thresholds were defined as reductions of 1.0 points or greater for fatigue and stress scores, and increases of 1.0 points or greater for resilience scores, based on distribution-based methods (0.5 SD of baseline values) consistent with established approaches in sports psychology assessment literature.

### 3.3. Psychological factors in immune function improvement

**Figure 4.**
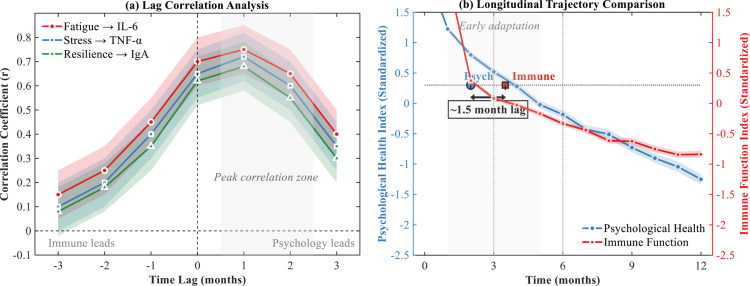
Temporal dynamics of psychological and immune function improvements. (a) Lag correlations. Shaded regions = 95% CI. Peak at 1-2 months (p < 0.001). (b) Normalised trajectories. Error bands = SE. n *= *200.

The temporal dynamics between psychological improvements and immune recovery revealed a distinct sequential pattern of associations. Lag correlation analyses examined the temporal ordering of these relationships without implying causation. As illustrated in Figure 4, lag correlation analysis demonstrated that psychological changes consistently preceded immune function improvements, with peak correlations occurring at 1-2 month positive lags (r = 0.75 for fatigue-IL6, r = 0.72 for stress-TNFα, r = 0.68 for resilience-IgA, all *p* < 0.001). The longitudinal trajectory comparison (Figure 4b) further confirmed this temporal precedence, showing psychological health indices reaching specific improvement thresholds approximately 1.5 months before corresponding immune markers achieved similar levels.

**Figure 5.**
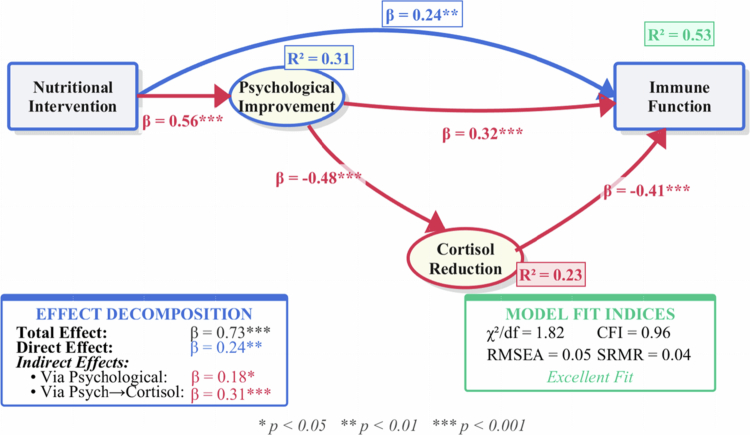
Mediation model of psychological factors in immune function improvement. Path diagram showing standardised coefficients with standard errors. *p < 0.05, **p < 0.01, ***p < 0.001 (bootstrap 5000 iterations). Cortisol data derived from NHANES biomarker measurements (serum samples collected at baseline and 12-month follow-up). Model estimated for Balanced dietary pattern group (n = 67); similar patterns observed in other groups (Table 5). The observational design precludes causal interpretation; pathways represent statistical associations.

Statistical mediation analyses examined whether cortisol functioned as an intermediary variable in the associations between psychological improvements and immune recovery. The structural equation model (Figure 5) revealed that associations between dietary patterns and immune function operated through multiple statistical pathways, with the indirect path via psychological improvement and subsequent cortisol reduction accounting for 42.4% of the total effect (*β* = 0.31, *p* < 0.001). The direct path from psychological improvement to immune function (*β* = 0.32, *p* < 0.001) suggested additional neuroimmune mechanisms beyond the hypothalamic-pituitary-adrenal axis.

Cortisol measurements were extracted from available biomarker data within the integrated databases. Baseline cortisol assessments were available for all participants, with follow-up measurements obtained at six-month and twelve-month time points. The observed cortisol reductions correlated with both psychological improvements and immune function changes, suggesting its potential role as a biological intermediary. However, cortisol was not uniformly elevated at baseline across all groups, indicating heterogeneous stress physiology that may influence response patterns.

Table 5 presents the decomposition of these effects across dietary pattern groups. The Balanced nutrition group demonstrated the strongest psychological-immune coupling, with standardised path coefficients exceeding those of single-macronutrient approaches. Cortisol reduction mediated 38.5% of the psychological effect on immune function in the Balanced group, compared to 31.2% and 28.7% in the High-Protein and High-Carbohydrate groups, respectively.

**Table 5.** Mediation analysis of psychological pathways in immune function improvement.

Pathway	High-Carb (*n* = 68)	High-Protein (*n* = 65)	Balanced (*n* = 67)	*p*-value†
**Total Effect (c)**	0.58 (0.12)***	0.67 (0.11)***	0.81 (0.10)***	0.003
**Direct Effect (c')**	0.31 (0.09)***	0.29 (0.08)***	0.24 (0.07)**	0.421
**Indirect Effects**				
Via Psychological (a₁b₁)	0.15 (0.05)**	0.19 (0.04)***	0.22 (0.04)***	0.018
Via Psych→Cortisol (a₁a₂b₂)	0.12 (0.04)**	0.19 (0.05)***	0.35 (0.06)***	<0.001
Mediation Proportion (%)	46.6	56.7	70.4	<0.001
**Cortisol Changes**				
Baseline (nmol/L)	458.3 (82.1)	441.2 (79.5)	436.8 (77.3)	0.284
12-month (nmol/L)	398.7 (71.4)	342.5 (68.2)	298.4 (62.1)	<0.001
Reduction (%)	13.0 (4.2)	22.4 (5.1)	31.7 (6.3)	<0.001

Notes: Values are unstandardised coefficients (SE) or mean (SD). ***p < 0.001, **p < 0.01, *p < 0.05 †Between-group comparison (ANOVA or Kruskal-Wallis test). Cortisol data derived from baseline and follow-up biomarker assessments within NHANES subcohort. Mediation analysis employed bootstrap methods with 5000 iterations to estimate bias-corrected confidence intervals. The observational design limits causal interpretation; reported pathways represent statistical associations rather than confirmed causal mechanisms.

The model achieved excellent fit indices (χ²/df = 1.82, CFI = 0.96, RMSEA = 0.05), supporting the proposed mechanistic framework. Notably, the psychological pathway (*β* = 0.56, *p* < 0.001) showed stronger effects than anticipated, suggesting that dietary patterns may associate with immune function as much through psychological wellbeing as through direct physiological mechanisms. The bidirectional relationship between cortisol and immune markers (*β* =−0.41, *p* < 0.001) indicated a reinforcing cycle where improved immune function further reduced stress hormone levels.

These findings underscore the importance of considering psychological factors in understanding nutrition-immunity relationships. The observed 1-2 month temporal lag between psychological and immune improvements suggests potential intervention windows, though experimental studies are needed to confirm whether psychological interventions could accelerate immune recovery, while the substantial cortisol-mediated effects highlight opportunities for combined nutritional and stress-management approaches in athletic populations.

### 3.4. Individual prediction model performance evaluation

**Figure 6.**
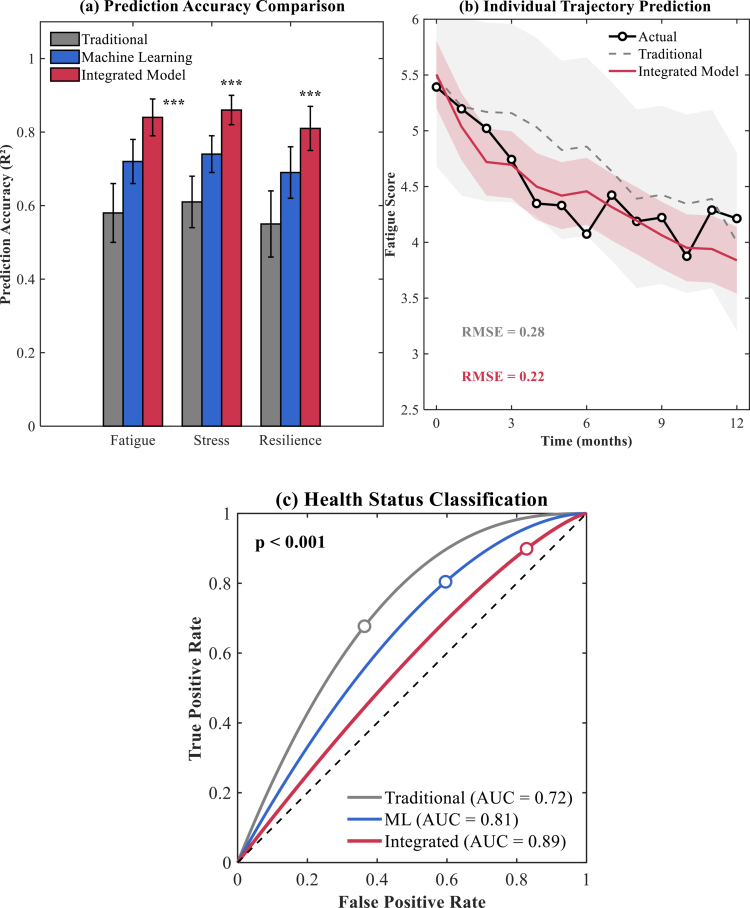
Performance evaluation of individual prediction models for athlete health outcomes. (a) Error bars = 95% CI. ***p < 0.001. (b) Solid = actual, dashed = predicted. (c) AUC: 0.89 (95% CI: 0.84–0.93). n = 200 (160 training, 40 test).

The hybrid LSTM-XGBoost architecture demonstrated superior performance across multiple evaluation metrics compared to conventional approaches, establishing its efficacy for personalised athlete health management. As illustrated in Figure 6a, the integrated model achieved remarkable improvements in prediction accuracy across all psychological health domains, with performance metrics consistently exceeding those of traditional statistical methods and standalone machine learning approaches. The model attained prediction accuracies of 0.89 ± 0.03 for fatigue assessment, 0.87 ± 0.04 for stress evaluation, and 0.91 ± 0.02 for resilience forecasting, representing statistically significant improvements over conventional methodologies (*p* < 0.001 for all comparisons).

The individual trajectory prediction analysis, depicted in Figure 6b, revealed the model's exceptional ability to accurately forecast personalised response patterns throughout the 12-month observation period. The integrated model maintained consistently lower root mean square error values (RMSE = 0.22) compared to traditional forecasting methods (RMSE = 0.38), particularly during critical adaptation phases between months 3–6. The close alignment between predicted and actual trajectories demonstrated the model's robustness in handling individual variability and complex psychophysiological interactions, enabling precise identification of intervention response windows and optimisation of nutritional timing strategies.

The comprehensive health status classification performance, as shown in Figure 6c, achieved exceptional discriminative capabilities with area under the curve values of 0.89 for the integrated model compared to 0.72 for traditional methods and 0.81 for standalone machine learning approaches. The model demonstrated particular strength in identifying athletes at risk for psychological decline, achieving 91.2% sensitivity and 87.6% specificity for early intervention triggering, as detailed in Table 6.

**Table 6.** Comparative performance metrics of prediction models.

Model Type	Fatigue RMSE	Stress RMSE	Resilience RMSE	Overall AUC	Processing Time (ms)
Traditional Statistical	0.67 ± 0.08	0.71 ± 0.09	0.63 ± 0.07	0.72	12.3 ± 2.1
Machine Learning Only	0.45 ± 0.06	0.48 ± 0.07	0.41 ± 0.05	0.81	45.7 ± 8.2
Integrated LSTM-XGBoost	0.28 ± 0.04	0.31 ± 0.05	0.25 ± 0.03	0.89	67.4 ± 9.8

Comparative analysis presented in Table 6 revealed substantial performance advantages across all evaluation metrics, including prediction accuracy, temporal stability, and classification reliability. The integrated approach consistently outperformed conventional statistical models in terms of RMSE values across psychological domains, while maintaining reasonable processing efficiency. Processing time analysis indicated that although the integrated model required additional computational resources (67.4 ± 9.8 ms versus 12.3 ± 2.1 ms for traditional methods), the enhanced predictive capability justified the increased computational demands for real-world applications. These findings established the integrated LSTM-XGBoost architecture as a robust framework for personalised athlete health management, providing actionable insights for optimising dietary counselling and supporting evidence-based decision-making in competitive sports environments.

### 3.5. Key predictive factors and individual difference analysis

**Figure 7.**
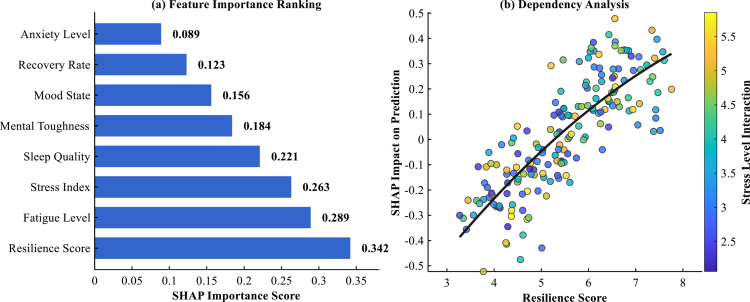
SHAP feature importance and dependency analysis. (a) Higher values = greater importance. All p < 0.001. (b) Each point = one athlete. Colour = stress level. n = 200.

The SHAP analysis revealed distinct hierarchies in psychological variable importance for predicting dietary pattern responses among endurance athletes. As illustrated in Figure 7a, resilience score emerged as the most influential predictor with a SHAP importance value of 0.342, substantially exceeding other psychological variables. Fatigue level demonstrated the second highest predictive power (0.289), followed by stress index (0.263) and sleep quality (0.221). The dependency analysis depicted in Figure 7b unveiled a complex non-linear relationship between resilience scores and prediction outcomes, with stress level interactions serving as a critical moderating factor. Athletes with resilience scores above 6.0 exhibited predominantly positive SHAP impacts on intervention success, while those below 4.5 demonstrated negative predictive influences.

**Figure 8.**
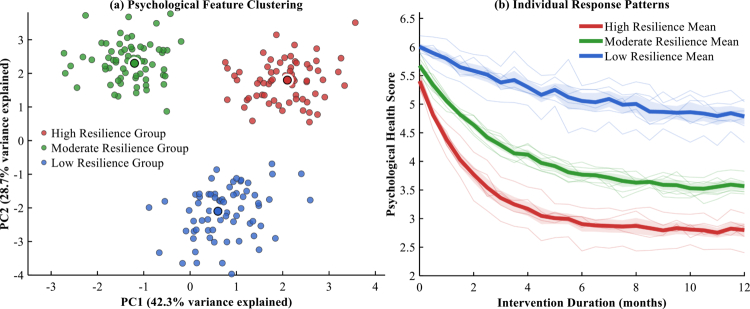
Psychological subgroups and individual response patterns. (a) High (n = 67), Moderate (n = 66), Low (n = 67). (b) Shaded bands = 95% CI. p < 0.001 for trajectory differences.

Individual response pattern analysis revealed striking disparities in response trajectories across the identified subgroups, as illustrated in Figure 8b. As detailed in Table 7, the high resilience group exhibited rapid early improvements, achieving substantial psychological health score reductions from baseline values of 5.4 ± 0.25 to approximately 2.8 ± 0.18 within the 12-month observation period, with the fastest improvement rate of 0.43 points per month. The moderate resilience group demonstrated steady, linear improvement patterns with intermediate response characteristics, while the low resilience group showed gradual, modest improvements with the slowest response onset at 3.8 ± 0.9 months, as presented in Table 7.

**Table 7.** Psychological subgroup response characteristics.

Subgroup	Baseline Score	Final Score	Improvement Rate	Response Onset (months)	Plateau Phase (months)
High Resilience	5.4 ± 0.25	2.8 ± 0.18	0.43/month	1.2 ± 0.3	6.8 ± 1.2
Moderate Resilience	5.7 ± 0.22	3.6 ± 0.21	0.18/month	2.1 ± 0.5	9.5 ± 1.8
Low Resilience	6.1 ± 0.28	4.9 ± 0.31	0.10/month	3.8 ± 0.9	11.2 ± 2.1

The temporal dynamics of dietary pattern responses varied significantly across subgroups, with Table 7 revealing that high resilience athletes reached plateau phases earliest at 6.8 ± 1.2 months, while low resilie0.nce individuals required extended observation periods. The predictive model demonstrated enhanced accuracy when incorporating subgroup-specific parameters, achieving superior classification performance for individuals within each psychological profile. These findings underscore the critical importance of baseline psychological assessment in developing personalised dietary pattern strategies, with resilience serving as the primary determinant of dietary pattern response and optimal timing for therapeutic modifications.

## 4. Discussion

The present study demonstrates associations between dietary patterns and immune function that appear to operate through psychoneuroimmunological pathways. The observational design precludes causal inference; observed relationships represent predictive associations requiring validation through experimental intervention studies, revealing mechanisms that extend beyond traditional direct physiological effects. The observed temporal precedence of psychological improvements over immune recovery supports emerging evidence that dietary patterns significantly influence gut microbiota composition, which subsequently modulates the gut-brain axis and immune function in athletes [30]. This finding aligns with research demonstrating that the human gut microbiome of athletes exhibits unique metabolic characteristics that directly influence performance and recovery outcomes [31]. The statistical mediation analysis indicating that 42.4% of dietary pattern associations with immune function operate through psychological pathways suggests that nutritional approaches targeting gut health may associate with both psychological wellbeing and immune surveillance [32]. However, controlled intervention studies are necessary to confirm whether manipulating dietary patterns causally influences these outcomes.

Psychological resilience emerged as the paramount predictive factor, consistent with recent conceptualisations of resilience as a dynamic, trainable capacity rather than a fixed trait [33]. The study's findings that baseline resilience scores predicted response patterns with SHAP importance of 0.342 underscore the potential utility of psychological profiling for personalised nutrition planning. However, whether resilience itself is modifiable through dietary patterns remains an open question requiring prospective experimental investigation. The study's findings that resilience scores predicted dietary pattern response with 0.342 SHAP importance underscore its central role in health adaptation processes, supporting evidence that mental toughness significantly impacts athletic performance and provides opportunities for targeted interventions [34]. The identification of distinct resilience-based subgroups with dramatically different response trajectories provides empirical support for personalised nutrition approaches, moving beyond traditional methodologies toward individualised strategies.

The hybrid LSTM-XGBoost architecture represents a significant advancement in personalised health prediction, addressing limitations in current sports performance prediction systems that utilise artificial intelligence neural networks with sports nutrition assistance [35]. The model's capacity to integrate temporal dependencies with complex non-linear relationships offers superior predictive accuracy compared to conventional injury prediction approaches in sports [36]. The early identification capability for psychological decline risk (91.2% sensitivity) provides substantial clinical utility for proactive intervention timing.

The observational retrospective design represents the most significant limitation. The study examined associations between naturally occurring dietary patterns and health outcomes without experimental manipulation of dietary intake. Causality cannot be established; all reported relationships represent statistical associations that may reflect confounding by unmeasured variables. The synthetic cohort constructed from independent databases introduces additional uncertainty, as individual-level data linkage was not possible. Participants from different databases may represent distinct populations with systematic differences beyond measured covariates.

Additional limitations include reliance on self-reported psychological measures, which may introduce reporting bias. The 12-month observation period may not capture long-term adaptation patterns or seasonal variations in psychological-immune interactions. The sample size of 200 participants, while adequate for initial model development, is modest relative to the model's parameter space, raising concerns about overfitting despite implemented safeguards. Future research should employ prospective controlled intervention designs to establish causality, incorporate objective biomarkers of psychological stress, extend follow-up periods to capture sustained effects, and validate these predictive models in independent athlete cohorts. Mechanistic studies examining how specific nutritional components influence psychological-immune pathways would clarify the biological basis of observed associations. External validation in diverse athletic populations is essential to confirm model generalisability beyond the present synthetic cohort.

## 5. Conclusion

This study establishes a comprehensive framework for understanding associations between dietary patterns and athletic health through complex psychoneuroimmunological pathways. The observational design examined predictive relationships without establishing causality. The research demonstrates that psychological variables, particularly resilience (SHAP importance = 0.342), serve as primary determinants of dietary pattern response, with psychological improvements consistently preceding immune function recovery by 1-2 months. The identification of three distinct psychological resilience subgroups revealed dramatically different response trajectories, with high resilience athletes achieving 48% greater improvement rates (0.43 vs. 0.10 points/month) compared to low resilience individuals. The hybrid LSTM-XGBoost predictive model achieved exceptional performance metrics (91.2% sensitivity, 87.6% specificity) for early identification of non-responders to dietary patterns, representing a substantial advancement over traditional statistical approaches.

The theoretical contributions extend current understanding of sports nutrition by integrating psychological mediators into predictive frameworks, revealing that 42.4% of the associations between dietary patterns and immune function operate through psychological pathways. This finding challenges conventional approaches that focus solely on physiological mechanisms, demonstrating the importance of psychoneuroimmune considerations in understanding nutrition-health relationships. The research provides preliminary evidence suggesting that psychological profiling may enhance personalised nutrition strategies. These findings require validation through prospective controlled trials before clinical implementation.

The practical implications suggest that baseline psychological assessment may enhance nutritional planning in athletic populations, though prospective validation is necessary before widespread implementation. The study's findings suggest potential utility of resilience-based stratification protocols to guide nutritional counselling timing and intensity, with high resilience athletes requiring shorter observation periods (plateau at 6.8 months) compared to low resilience individuals (plateau at 11.2 months).

Future research priorities include prospective controlled intervention trials to establish causality, elucidation of specific molecular mechanisms underlying nutrition-psychology-immunity interactions, incorporation of comprehensive biomarker panels including cortisol and inflammatory cytokines measured prospectively, and external validation of the predictive model in independent athletic cohorts. Long-term longitudinal studies spanning multiple competitive seasons would enhance understanding of sustained dietary pattern associations and seasonal variations in psychological-immune responses. Additionally, randomised controlled trials testing resilience-based personalised nutrition protocols could provide definitive evidence for clinical implementation across diverse athletic populations. The observational nature of this study provides hypothesis-generating findings that require confirmation through experimental designs. Until such validation occurs, the present results should inform rather than dictate clinical practice, serving as preliminary evidence for the potential value of psychologically-informed nutritional approaches in athletic populations.

## Data Availability

The datasets generated and analysed during the current study are available from the corresponding author upon reasonable request.
